# Marine snow morphology illuminates the evolution of phytoplankton blooms and determines their subsequent vertical export

**DOI:** 10.1038/s41467-021-22994-4

**Published:** 2021-05-14

**Authors:** Emilia Trudnowska, Léo Lacour, Mathieu Ardyna, Andreas Rogge, Jean Olivier Irisson, Anya M. Waite, Marcel Babin, Lars Stemmann

**Affiliations:** 1 Institute of Oceanology Polish Academy of Sciences, Sopot, Poland; 2 Takuvik Joint International Laboratory (CNRS and Université Laval), Québec, QC Canada; 3 Department of Earth System Science, Stanford University, Stanford, CA USA; 4 Sorbonne Université, CNRS, Laboratoire d’Océanographie de Villefranche, LOV, Villefranche-sur-Mer, France; 5 Institute for Ecosystem Research, Kiel University, Kiel, Germany; 6 Alfred Wegener Institute, Helmholtz Center for Polar and Marine Research, Polar Biological Oceanography Section, Bremerhaven, Germany; 7 Ocean Frontier Institute and Department of Oceanography, Dalhousie University, Halifax, Nova Scotia Canada

**Keywords:** Carbon cycle, Marine biology

## Abstract

The organic carbon produced in the ocean’s surface by phytoplankton is either passed through the food web or exported to the ocean interior as marine snow. The rate and efficiency of such vertical export strongly depend on the size, structure and shape of individual particles, but apart from size, other morphological properties are still not quantitatively monitored. With the growing number of in situ imaging technologies, there is now a great possibility to analyze the morphology of individual marine snow. Thus, automated methods for their classification are urgently needed. Consequently, here we present a simple, objective categorization method of marine snow into a few ecologically meaningful functional morphotypes using field data from successive phases of the Arctic phytoplankton bloom. The proposed approach is a promising tool for future studies aiming to integrate the diversity, composition and morphology of marine snow into our understanding of the biological carbon pump.

## Abstract (web summary)

Marine snow is a major route through which photosynthetically fixed carbon is transported to the deep ocean, but the factors affecting flux are largely unknown. Here the authors use high frequency imaging of marine snow particles collected during phytoplankton blooms to categorize and quantify transport.

## Introduction

One key vector of the biological carbon pump is marine snow, representing large (>500 µm) particles of detritus (including zooplankton fecal pellets), organic matter (remnants and exudates of plankton), inorganic matter, and/or aggregated mixtures of those^1–5^. Changes in both phytoplankton and zooplankton community structure directly influence the quantity and composition of marine snow^6,7^ and thus the efficiency of the biological carbon pump^8,9^. Many previous biogeochemical models^5,10,11^ and calculations estimating carbon export, based on in situ observations^12–14^, assumed a fixed mass to volume relationship and simple geometry of particles, such as spheres or a defined fractal dimension. This simplification was necessary because marine snow is difficult to study due to its fragile nature and complex morphological characteristics. However, the role and fate of marine snow in vertical particle flux are strictly determined by its key features, including morphology (size, shape, porosity) and composition (organic, mineral). These features have an impact on aggregation and disaggregation processes, export rate to the deep sea, and biological interactions (colonization, feeding) with the plankton community^2,15–17^. Therefore, an objective and universal method to classify marine snow into functional types represent an important step forward to understand and follow the pathways of the biological carbon pump^4,8,18–20^, and the deposition of e.g., microplastics^21^. Although the capacity to detect and enumerate marine snow in situ has radically improved with the development of optical methods^22–24^, the establishment of distinct, standardized morphological categories of marine snow remains a methodological challenge due to their high numbers, vast variety, and smooth transitions between individual classes. Over the last 10 years, various optical systems have provided millions of images of plankton and marine snow, with an average ratio of 1 organism per 100 particles^18,25,26^. Some studies have focused on plankton images, which are less abundant and can be classified into distinct morphological groups^25,27,28^. The ability to discriminate between ecologically relevant groups of marine snow is still poor^29^, even though it may be critical to incorporate their morphological information into studies of the spatio-temporal patchiness in plankton production and its subsequent export via the biological carbon pump^24,30^.

**Fig. 1:**
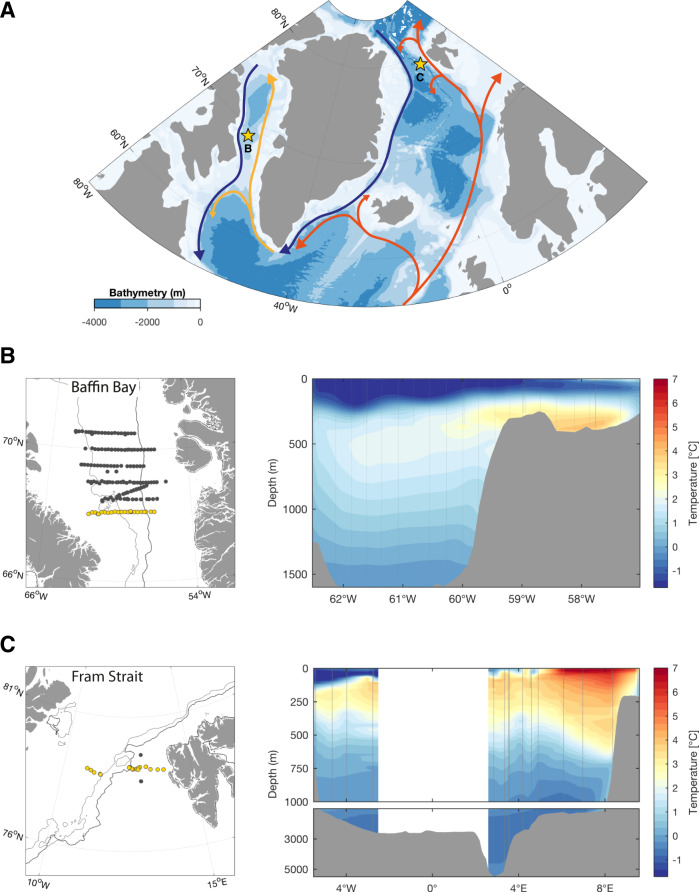
Study areas: the Baffin Bay and Fram Strait. **A** Station locations (stars) with a schematic representation of the main ocean currents (arrows, blue: outflow of Arctic water masses, yellow and orange: inflow of Atlantic water masses). The close-up maps of the particular regions (**B**: Baffin Bay, **C**: Fram Strait) present the sampling stations (yellow dots indicate stations used for the associated temperature sections). The mean 15% sea ice concentration limit of the ice edge for June and July are indicated by dark and light gray contour lines, respectively.

Here we propose an approach in the processing of underwater images collected by the Underwater Vision Profiler, which database is already extremely large globally (tens of millions of images) and growing exponentially. We apply statistical discrimination of marine snow into morphotypes and study the linkages between their composition in relation to the dynamics of plankton communities on the example of two comparable study areas within the Arctic marginal ice zones (the Baffin Bay and Fram Strait, Fig. 1) which are characterized by high productivity^20,31,32^ and distinct phytoplankton bloom phases^19,33,34^. We hypothesized that our method could be used to investigate how the concentration of different categories of marine snow and their vertical distribution change during sequential phases of the phytoplankton bloom and how this could be linked to the carbon export. We compare qualitatively resolved patterns with more classical analyses including total concentration, biovolume, and size distribution of marine snow. We further discuss the potential of marine snow classification applied to underwater imaging techniques in order to integrate an ecological approach for a better understanding of the ocean’s carbon cycle.

## Results

### Morphotypes of marine snow

**Fig. 2:**
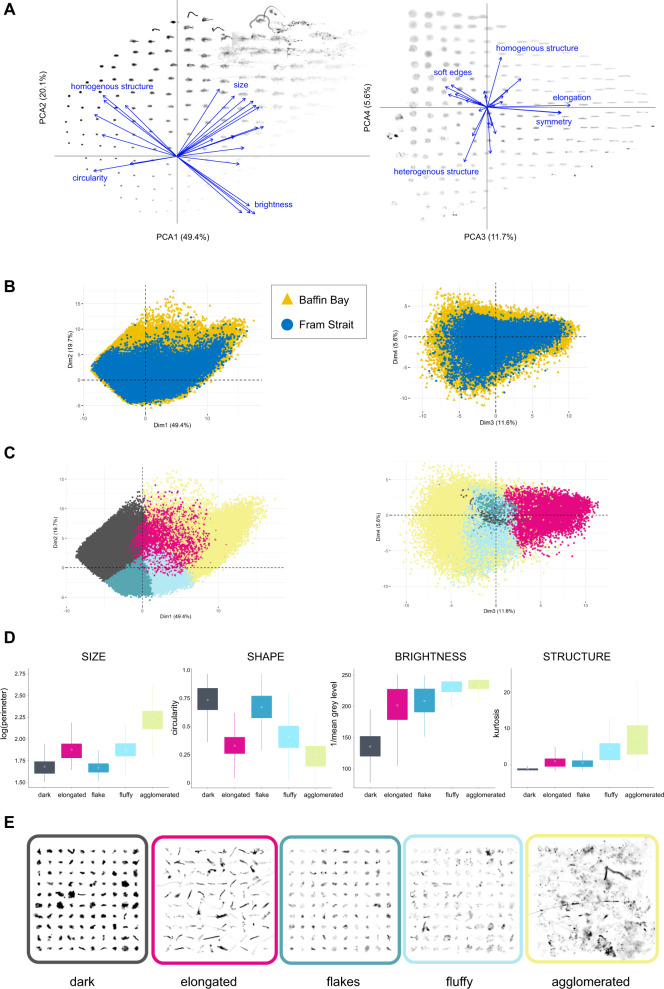
Assignation of marine snow into morphotypes. **A** The PCA morphospace presenting correlations between 24 morphological characteristics of individual marine snow particles (arrows) and their exemplary appearance spread over the 1st and 2nd axes on the left and the 3rd and 4th axes on the right side. **B** The projection of 1,063,576 marine snow particles in the common morphospace of two sampling regions distinguished by color (blue: Fram Strait, yellow: Baffin Bay). **C** Categorisation of the marine snow into 5 morphotypes by k-means clustering (color-coded). **D** Box plots of the median values (size of the boxes present 25th and 75th quartiles, whiskers present distance from the quartile max/min to 1.5 * IQR (inter-quartile range)) of key exemplary morphological properties (perimeter, circularity, 1/grey level, kurtosis of grey level) characterizing 5 morphotypes. **E** Representation of example images of marine snow in each category morphotype.

Twenty-four morphological properties of individual marine snow objects (Supplementary Table 1) representing their size (e.g., area, perimeter), shade intensity (e.g., mean/median gray level), shape (e.g., symmetry, elongation), and structure (i.e., homogeneity or heterogeneity, mostly based on the variability in gray level) were combined to build a PCA (Principal Component Analysis) space (Fig. 2). The k-means clustering applied to the PCA coordinates defined five marine snow morphotypes. Type 1 comprised dark objects, that are mostly small (the mean perimeter of 50 pixels ~4 mm), circular and homogenous (Fig. 2; Supplementary Table 1). Type 2 consisted of elongated objects, that are medium-sized (the mean perimeter of 78 pixels ~7 mm) and quite diverse in their brightness (Fig. 2). Type 3 consisted of flake-type marine snow, represented by the small (the mean perimeter of 46 pixels ~4 mm), circular, but bright marine snow. Type 4 represented fluffy flocs, that were medium-sized (the mean perimeter of 75 pixels ~7 mm), bright, and had heterogeneous forms. Type 5 consisted of large (the mean perimeter of 194 pixels ~17 mm), bright, agglomerated forms of multielement structure (Fig. 2, Supplementary Table 1). These five morphotypes were then used to characterize the distribution and composition of marine snow.

### Sea ice-related time series of marine snow morphotypes–Baffin Bay

In the Baffin Bay, the dynamics of the distribution of specific morphotypes of marine snow were analyzed according to Open Water Days (OWD), which aligns stations in relation to ice conditions. OWD indicates how long the station had been ice-free before sampling (positive values) or how long after sampling it took to become ice-free (negative value).

The first signal of elevated concentrations of marine snow was observed under the ice (−20 OWD) and significantly increased around the time of the ice break-up (0 OWD) until reaching the maximum after 20 days of ice-free conditions (Supplementary Fig. 1). Increased sizes of marine snow were detected 15 days after sea ice break-up below depths of 100 m, which were also associated with less steep size spectra slopes (Supplementary Fig. 1).

**Fig. 3:**
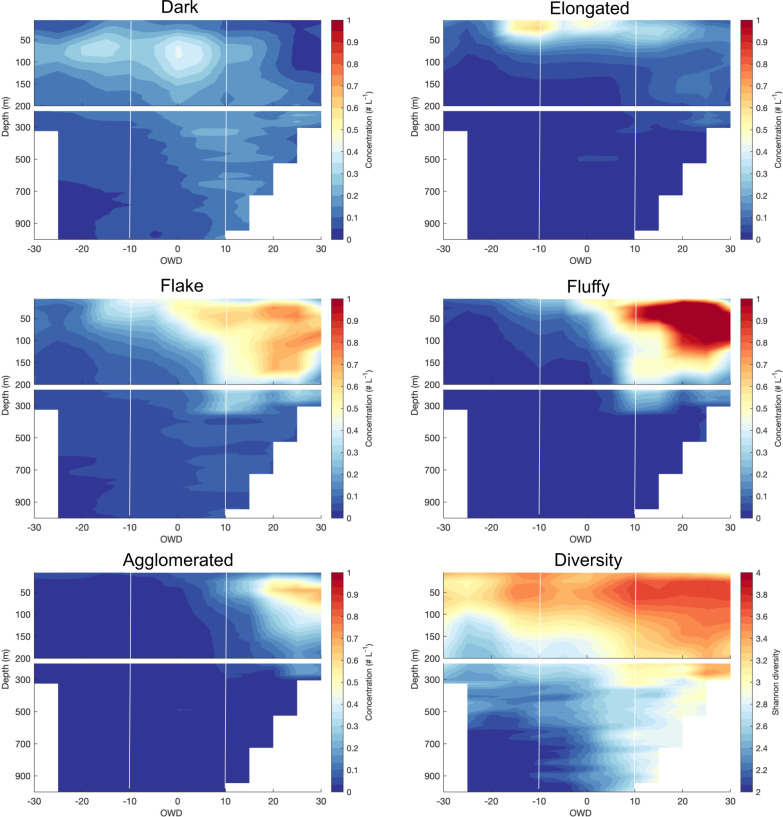
Spatio-temporal distribution of marine snow morphotypes. Section plots of the concentration of various morphotypes of marine snow, their diversity (Shannon-Wiener index) over depth and time (open water days (OWD)) in the Baffin Bay. White vertical lines indicate the ranges of assigned periods (under ice, ice break-up, ice-free).

The concentration maximum of dark compact marine snow was located between 50 and 100 m of the water column before sea ice break-up, and it deepened successively afterward down to ~1000 m (Fig. 3). Throughout the observation period, dark compact particles’ vertical attenuation was the lowest of all marine snow categories, with visibly more dark compact morphotypes in deep waters at the end of the bloom (Supplementary Fig. 2). This type of marine snow-dominated below 100 m until 10 OWD (Supplementary Fig. 3), but later on they were outnumbered by other morphotypes (Supplementary Fig. 3). The highest concentration of elongated forms of marine snow was found in the upper 50 m layer throughout the investigated period, whereas a distinct peak could be observed at −10 OWD and during sea ice break-up (Fig. 3, Supplementary Fig. 3). Their vertical attenuation was the highest among all the marine snow morphotypes but decreased rapidly after OWD = 10 (Supplementary Fig. 2). Flake and fluffy forms of marine snow were two the most abundant morphotypes in the upper 200 m of ice-free waters (>0 OWD), but rare under ice-covered conditions (Fig. 3). The concentration maximum of large heterogeneous agglomerated forms of marine snow was observed at the late ice-free phase (>10 OWD), where they were mainly located in subsurface waters between 20 and 100 m (Fig. 3). The highest diversity (Shannon-Wiener index) of marine snow composition was observed within the upper 100 m until ice break-up, after which it increased also in deeper waters (300 m before and 500 m after 20 OWD, Fig. 3).

### Sea ice-related time series of the coupling between phytoplankton, marine snow, and zooplankton–the Baffin Bay

**Fig. 4:**
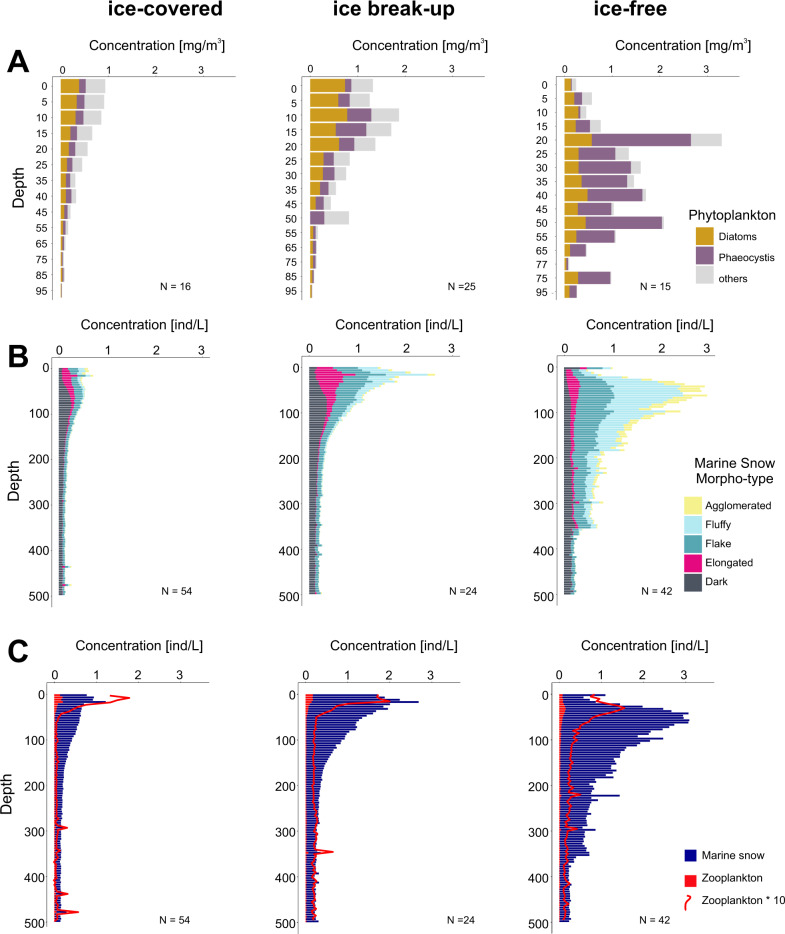
Coupling between phytoplankton, marine snow morphotypes, and zooplankton among periods over defined sea ice coverage phases: ice-covered (pre-bloom), ice break-up (bloom), ice-free (late bloom) in the Baffin Bay. **A** Pigment concentrations of dominant phytoplankton groups. **B** cumulative concentrations of different marine snow morphotypes. **C** total concentrations of marine snow and zooplankton. The red line highlights the distribution of zooplankton (the concentration multiplied by 10).

The ice-covered period was characterized by particulate matter containing a relatively low concentration of phytoplankton pigments (<1 mg m^−3^), consisting mainly of diatom origin (Fig. 4A), and also by a low concentration of marine snow, mainly consisting of dark, elongated, and flake morphotypes (Fig. 4B). With the ice break-up, the concentration of phytoplankton pigments increased, and diatoms remained the dominant group (Fig. 4A). At that time, a pronounced (>0.5 ind.L^−1^), but the narrow peak of elongated marine snow, restricted to the upper 20–30 m layer appeared (Fig. 4B). Under ice-free conditions, the contribution of diatoms decreased, while *Phaeocystis* bloomed, mainly below 20 m (Fig. 4A). This change in the dominant group of phytoplankton was accompanied by a change in the composition of marine snow, which at that time was the most variable among the stations (Supplementary Fig. 4). Dark and elongated morphotypes, which dominated under the ice and at the ice break-up, were outnumbered in the ice-free waters by the fluffy and agglomerated morphotypes (Fig. 4B).

The concentration of marine snow drastically exceeded zooplankton concentrations throughout the study (Fig. 4C). During the ice-covered and ice break-up phases, the highest zooplankton concentrations were observed near the surface, while they were most abundant at 30 m depth during ice-free conditions (Supplementary Fig. 5).

### Spatio-temporal coupling between phytoplankton, marine snow, and zooplankton–Fram Strait

**Fig. 5:**
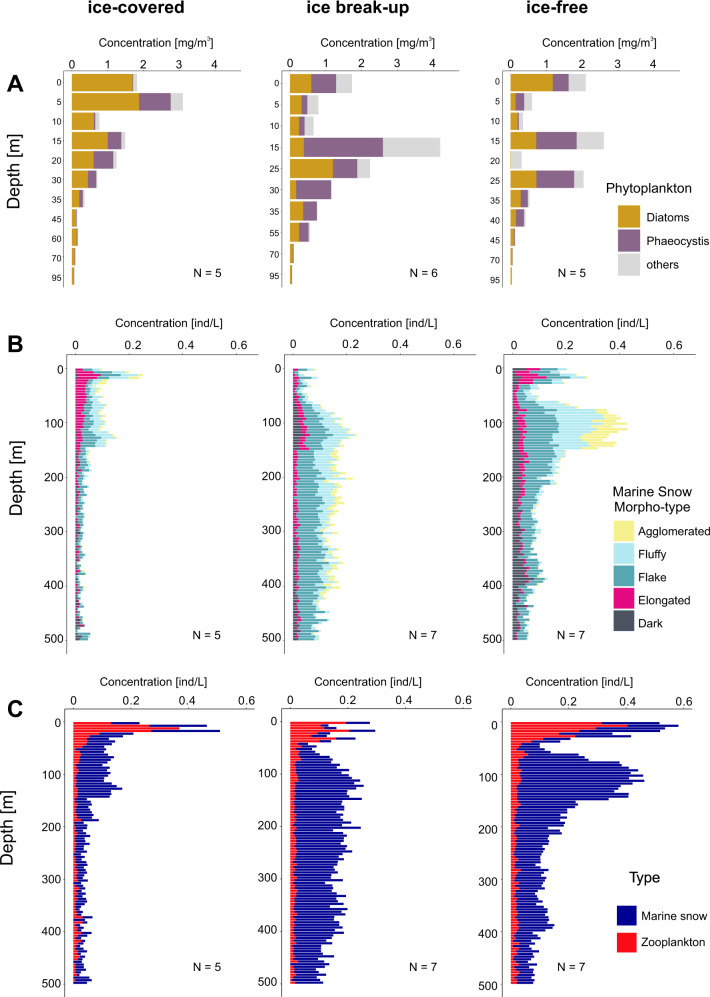
Coupling between phytoplankton, marine snow morphotypes, and zooplankton among periods over defined sea ice coverage phases in Fram Strait. **A** Pigment concentrations of dominant phytoplankton groups. **B** Cumulative concentrations of the different marine snow morphotypes. **C** Total concentrations of marine snow and zooplankton.

In Fram Strait, diatom pigments dominated in the ice-covered phase, and they co-occurred with *Phaeocystis* during the ice break-up and ice-free conditions. Elevated pigment concentrations (>1 mg.m^−3^) of diatoms were observed near the surface, whereas *Phaeocystis-*type pigments were observed below 15 m (Fig. 5A). Generally, low marine snow concentration, dominated by elongated morphotypes, was observed under the sea ice and concentrated mainly in the upper 150 m (Fig. 5B). Interestingly, fluffy and agglomerated marine snow was rather rare but already present under the ice (Fig. 5B). At ice break-up, high concentrations of phytoplankton pigments were accompanied by high concentrations of zooplankton in the upper 50 m, but not by an increase of the marine snow concentrations (Fig. 5C). In contrast, high concentrations of marine snow (appr. 0.4 ind.L^−1^), dominated by flake and fluffy types, were found below the 50 m surface layer extending substantially over depth down to 500 m (Fig. 5B), but with high intra-station variability (Supplementary Fig. 4). At the ice-free stations, the still relatively high (>0.5 mg m^−3^) concentration of diatom chl *a* (Fig. 5A) corresponded to the surface peak dominated by dark and elongated morphotypes (appr. 50%) in the upper 50 m (Fig. 5B), concurrent with the zooplankton concentration maximum (Fig. 5C). The second peak of marine snow was restricted to between 50 and 150 m, consisting of a mixture of flake, fluffy, and agglomerated morphotypes. In general, zooplankton contributed a lot to images collected by Underwater vision profiler (UVP) within the upper 50 m of the water column in Fram Strait (Fig. 5C).

## Discussion

Since the pioneering work of Alldredge and Silver^1^ it has been widely recognized that marine snow consists of aggregated particles of diverse size and origins, mainly produced by plankton and brought together by different biological and physical processes^18^. The extreme variability in their nature, size, porosity, elemental composition, and spatio-temporal dynamics makes it a huge methodological challenge to observe and quantify them objectively. Previous studies that applied marine snow catchers^35,36^, gel-filled sediment traps^37,38^ in situ, or mesocosm experiments^7^ revealed essential features of marine snow morphological attributes and biogeochemical content. However, those investigations presented a rather low spatial or temporal resolution, as could focus only on a limited amount of objects. Other in situ sensors such as laser counters^39,40^, coulter counters^41,42^, or cameras^22,24^ have been used extensively to address spatial and temporal particle dynamics, but without taking their structural properties into account, aggregating all morphotypes of marine snow in one detritus compartment resolved by size^18^. Although size is not always directly correlated with the sinking velocity of marine snow^43^, most studies were neglecting other morphological attributes. Here we present that advanced in situ imaging techniques enable us to analyze also many other morphological properties of marine snow, including their shapes, darkness, and structural heterogeneity.

To do this, we created an approach for the robust classification of marine snow into several ecologically meaningful categories, based on a set of individual morphological attributes of single marine snow images. We then questioned whether such qualitative marine snow analysis could provide a better understanding of particulate dynamics during different stages of phytoplankton blooms than by analyzing size-related metrics alone.

The classification of marine snow into morphotypes was based on several methodological choices available from the many possible options considered. Our choices were motivated partly by the practicality of the computation (with millions of objects) and furthermore by the interpretability of the results. And so the decision fell on the number and type of morphological properties (24 properties presented in Supplementary Table 1), PCA as the dimension reduction, and k-means as the clustering algorithm. For example, we chose a rather low number of categories (five: dark, elongated, flake, fluffy, and agglomerated) to facilitate ecological interpretation, while retaining enough detail and avoiding hindering the perception of compositional change. Defining categories of marine snow is even more difficult and less objective than for plankton taxa, because of the continuity in their morphological attributes and the infinite variability of forms that may be embodied. Therefore, no classification scheme will be able to create groups of well-defined types of marine snow that all look alike; rather they will create groups that each contain a fairly wide variety of forms but that still share some common characteristics. Here, Arctic phytoplankton bloom and its successive phases in the Baffin Bay gave us an initial context to train the classification scheme and assess its scientific relevance. The data from Fram Strait was used to test our classification scheme and its ecological interpretation within an analogous process. Inter-rater reliability analysis showed that the classification into morphotypes was very robust to changes in the dataset used.

The origin of specific morphotypes of marine snow was determined based on both a priori knowledge of the bloom dynamics in studied regions and on the concurrent datasets of oceanographic variables related to the ice retreat and phytoplankton composition (HPLC, microscopy).

The potential origin of the dark morphotype can be very broad. In the upper layers of the ice-covered stations, they were formed in association with ice algae, which, when accumulated under the sea ice^44–47^ tend to aggregate and become metabolically less active^48^. During sea ice break-up, the release of ice algae into the underlying water increased the concentration of this morphotype rapidly (Fig. 3). Their constant presence, regardless of the phytoplankton bloom phase and depth layer (Figs. 4 and 5), indicate that they may also represent other objects such as fecal pellets and/or fragments of such and larger aggregates^15,36^. As they were a dominant morphotype of marine snow in the deeper layers, they are likely also to represent old^49^, fragmented or densified objects, as compactness is increasing with depth^50^. Even though marine snow undergoes a myriad of transformations when settling down, our observations show that it mostly ends up in form of the dark morphotype in the deep ocean.

The distribution of the concentration peaks of dark morphotype was clearly associated with the steepest size spectra slopes (indicating the prevailing role of the smallest objects) from the surface to 900 m depth (Fig. 3 and Supplementary Fig. 1). Assuming they were produced in large quantities at ice break-up at the surface and observing them at a depth of 900 m 10 days later, an estimated maximum sinking speed of about 90 m.d^−1^ can be inferred^23^. This clearly shows how the signal of spatio-temporal patchiness in plankton production may be efficiently transferred to the deep and form the ‘particle injection pump’^4^. A type I linear regression of time of the maximum concentration signal (dependent variable) vs. depth (independent variable) yielded an average sinking rate estimate of 38 m.d^−1^ (Supplementary Fig. 6)). This rough estimate is of the same order of magnitude as sinking speeds calculated using similar optical methods during the spring bloom in the Mediterranean Sea (estimated as 30 and 40 m d^−1^ for 0.5–1 mm and >1 mm aggregates, respectively)^51^ and lower than the rate of export pulses during the evolution of the sub-polar North Atlantic spring bloom (~74 m d^−1(^^52^^)^), or episodic sedimentation events in the upwelling system off California (mean 75 m d^−1^, median 59 m d^−1(^^53^^)^).

The source of the elongated morphotypes is also difficult to discriminate explicitly. Their appearance is similar to diatom chains and indeed they matched with the presence of diatoms either under the ice in Fram Strait^54^ or at the ice break-up in Baffin Bay^55^ (Supplementary Fig. 2). They may also embody fecal pellets, as their distribution was spatially aligned with the zooplankton distribution (Supplementary Fig. 5), especially in Fram Strait (Fig. 5), where the younger life stages of *Calanus* spp. are dominant at that time^56,57^ and could benefit from the bloom by intensive feeding. The elongated morpho-types can also represent colonies of phytoplankton, or various filaments, or tentacles of cnidarians. Those elongated forms apparently do not sink efficiently, since they were found mostly in the upper 100 m. Sinking speeds calculated following the depth distribution and time of their peaks (average of 7.2 m d^−1^ Supplementary Fig. 6) are much lower than the sinking rates of dark morphotypes. It is possible that they are not settling as elongated forms, but become fragmented into the dark categories or aggregated into the flake, fluffy, or agglomerated morphotypes (starting to accumulate at ice break-up and peaking in ice-free phase) (Fig. 3; Supplementary Fig. 2).

Flake and fluffy morphotypes are representatives of typically perceived “marine snow”, appearing as rather circular and light flocks. They were numerically dominant in ice-free waters of both Baffin Bay and Fram Strait during the late bloom phase, which was associated with the accumulation of phytoplankton biomass^58^ and change in marine snow composition^55,54^. Flake-type marine snow can be the precursors of the fluffy forms, which are created when the bloom is massive enough to enhance aggregation. These, together with the large heterogeneous agglomerated category (peaking in ice-free conditions) were associated with the increasing importance of *Phaeocystis* as the bloom progressed (Supplementary Fig. 5). The formation of agglomerated morphotype of marine snow was likely greatly enhanced by exopolymeric polysaccharides produced by *Phaeocystis* when forming mucilaginous colonies^50,59^. The increased stickiness of such aggregates can play a ‘cleaning’ role in the water column, by scavenging smaller particles while settling^2,60,61^, resulting in a large size and heterogeneous structure^62^. This may be the reason why the *Phaeocystis*—origin marine snow could also be found at great depths. The short time span of their observation late in the bloom prevented the estimate of their settling speed as for the two other categories (Supplementary Fig. 6). However, all three categories developed their sub-surface peak at 50 m after ice break-up and then extended to 300 m depth during the late bloom phase in ice-free waters with a strong depth attenuation suggesting their slow settling. Because the dark morphotype, which is mostly small and compact had a five times higher estimated sinking speed than the larger elongated type (Supplementary Fig. 6), we can conclude that size is not always the dominant factor driving particle settling speed and that composition, compactness, and density have also to be taken into account^43^. Such morphologically-driven differences in settling speeds impact the vertical attenuation of marine snow particles, which can be summarized as the exponent “b” in the Martin Curve^63^. The dark morphotype showed the lowest attenuation, while the elongated forms had the highest values (Supplementary Fig. 2). By contrast, *b* values calculated from the vertical profile of total concentrations were more stable over time and did not reflect the high variability between morphotypes. The fact that the attenuation of specific morphotypes differed from the average is a further argument pointing towards the additional value of marine snow classification into specific categories. The observed temporal pattern of each morphotype (the appearance and increasing/decreasing trends observed in Fig. 3) was consistent with the temporal pattern of each group’s vertical attenuation and the resulting concentrations at 100 m (N_100_ observed in Supplementary Fig. 2). According to our results the composition of marine snow may change clearly over depth (e.g., the shift from the diversified composition in the upper 100–150 m to the clear dominance of dark morphotype in the deepest layers). All these indicate that our method is a major step forward in our capacity to depict complex marine snow dynamics compared to the more classical, size-based approach. Thus, developing a generic classification approach and improved algorithms to calculate mass and flux, which take also morphological properties into account will be promising tools for our understanding and quantification of the biological carbon pump.

**Fig. 6:**
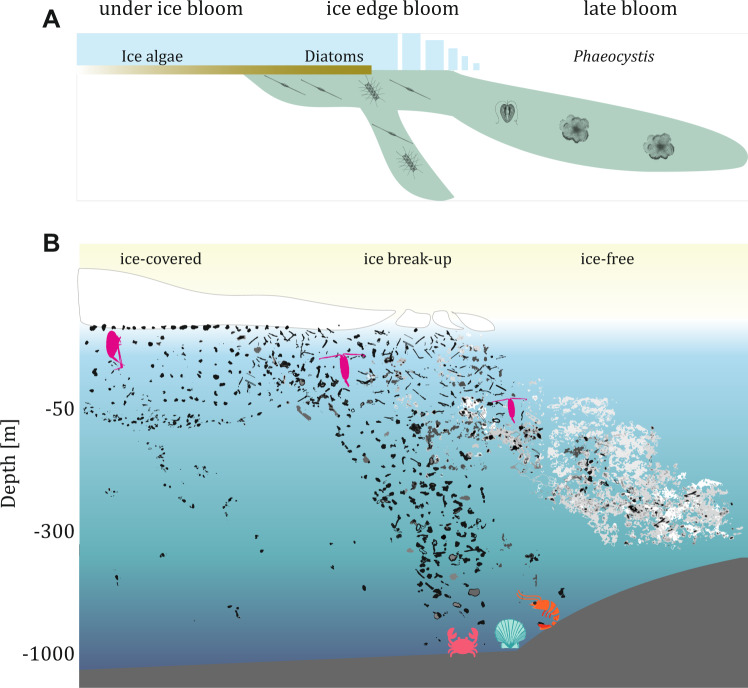
Conceptual schemes of the phases of the Arctic ice-associated phytoplankton bloom dynamics extended by the corresponding morphological changes of marine snow. The exemplary marine snow images assessed by the UVP (Underwater Vision Profiler) are presented over various phases of the Arctic phytoplankton bloom. **A** Conceptual scheme of the stages of the Arctic phytoplankton bloom. **B** Conceptual scheme of the corresponding modification in marine snow composition.

In both the Baffin Bay and Fram Strait, the classical Arctic bloom succession, from diatom-dominated to flagellate-dominated communities^59,64–67^, was clearly accompanied by changes in the morphology and composition of marine snow. We therefore can extend the traditional scheme of the Arctic phytoplankton bloom phenology (Fig. 6A) with a conceptual scheme of the developmental phases of marine snow composition (Fig. 6B).

By following the changes in marine snow morphology, we could observe that the first, sea ice-covered phase, was characterized by low concentrations and thus low export of marine snow, mainly composed of the dark, compact morphotype in Baffin Bay and elongated forms in Fram Strait. At the time of sea ice break-up, not only increased marine snow concentration but also diversity could be observed (Fig. 6). Although previous studies suggest that the diversity of marine snow is rather low (e.g., 95% represented by the same type^68^), the bloom at the ice break-up phase was accompanied by a mixture of many categories of marine snow. In the late bloom phase that was observed in ice-free waters, large agglomerated forms of marine snow were observed in both campaigns in the form of massive sub-surface peaks. The relatively deep location of concentration peaks of those heterogeneous agglomerates points towards their effective sinking or towards their association with Atlantic waters^69,70^, which lay below the surface, Polar Waters. Because in both study regions, the succession of phytoplankton was connected with different hydrographical regimes: the observed differences in marine snow composition may also reflect the differences in the ecological states of particular water masses^58,54,71,72^.

With the increasing number of in situ imaging technologies, there is an urgent need for classification methods for marine particles and plankton. Plankton classification has been developed in numerous studies, both for the development of classification algorithms^29,73–75^ and for retrieving ecological dynamics from imaging datasets^76–79^. In contrast, marine snow classification has not been addressed quantitatively, although analysis of marine snow dynamics remains fundamental for building an understanding of trophic interactions and carbon sequestration. Our proposed classification of marine snow into several ecologically meaningful morphotypes represents a useful approach to better understand sources and sinks of plankton material and provides data about structural features of marine snow that would not be possible using size distribution analysis. Direct and immediate digitalization of particles and plankton, accompanied by computational methods for object classification enables fast data processing, which may assist in defining sampling strategies during cruises in almost real-time. It also opens a new avenue for monitoring the high-frequency spatio-temporal patchiness of the pelagic realm by autonomous devices, such as moorings and sea gliders^80^.

However, unlike plankton classification, which benefits from externally-defined taxonomy, with clear distinctions between taxa, the classification of marine snow must address a continuous variation of properties and encompass a mixture of diversely sourced particles. Furthermore, we cannot make explicit recognition of the origin (content) of individual marine snow, since they are not collected. But in turn, one may set a question whether we actually need to know it. Developing new algorithms calculating sinking speed by considering not only size but also image-derived porosity or density would be a considerable improvement in estimating flux from in situ images, which, for the moment, solely relies on size^24,30^). Furthermore, we consider that similar to what was recognized for plankton^79,81,82^, the morphological trait-based approach should be informative and complementary to other approaches to assess and model a variety of ocean processes. Moreover, one important advantage of the classification we proposed is that it is based on deterministic morphological features, easily assessed and compared across systems. The challenge relies on building training sets representative of most existing morphotypes. This challenge may be resolved by an augmented observation effort^22,83^.

At this state, the typology we propose is only valid for ecosystem dynamics restricted to a given time and place (phytoplankton bloom at the Arctic ice edge). The high degree of compatibility between morpho-spaces created for two independent datasets suggests that the addition of new data from blooms with similar phytoplankton succession in other open ocean Arctic regions should not radically change the classification system. However, adding new datasets from other ecosystems in the Arctic (e.g., fjords submitted to mineral particle injection from glaciers and rivers), other locations across the world (e.g., tropical regions dominated by picophytoplankton), or other seasons would likely allow the identification of more categories of marine snow. Although we have not captured the full range of possible marine snow morphotypes present in the oceans, the ability to distinguish them is evident in our results and is a promising avenue for a worldwide marine snow classification based on underwater images. A future compilation of worldwide UVP datasets would provide better insight into how many combinations of morphotypes can be distinguished and interpreted globally and whether marine snow differs drastically among regions, or is rather uniform because it undergoes similar modification processes. As the modifications of marine snow morphology are so spectacular over time and depth, one ultimate perspective resulting from the use of the method presented would be the definition of a ‘transformation function’ between various morphotypes.

Because billions of images will be collected in the coming years, the extensive list of potential scientific outcomes enabled by this method should motivate future efforts to further develop and implement objective marine snow classifications. The possibility to gain qualitative information on each individual marine snow particle in its natural environment, to identify it, and to retrieve its ecological role in trophic webs and carbon sequestration is the most important step forward we have taken. For future studies, we recommend broadening the perspectives of the selected morphotypes interpretations, extending the ‘taxonomical’ catalog of possible types of marine snow, and expanding it over other regions and processes.

## Methods

### Sampling locations

Two separate research programs were conducted in June–July 2016.

As part of the GreenEdge project, the CCGS Amundsen icebreaker sampled 164 stations along east–west transects by following the sea-ice retreat in the Baffin Bay (Fig. 1). The Baffin Bay is a deep-water basin connected to the Arctic Ocean through three straits: Lancaster Sound, Jones Sound, and Nares Strait. It is covered by sea ice during winter; it becomes completely ice-free only in July. Between these two situations, there is a marginal ice zone present in this area for most of the year. The Baffin Bay is characterized by intense hydrographic gradients caused by interactions between the northward-flowing relatively warm current from the North Atlantic (West Greenland Current) and the colder southward flowing current from the Arctic (Baffin Current)^58^ (Fig. 1).

As part of the FRAM project (Frontiers in Arctic marine Monitoring, Long-Term Ecological Research LTER Hausgarten), Fram Strait was sampled at 26 stations (Fig. 1) from the RV Polarstern. Fram Strait is the only deep gateway into the Arctic basin and a crucial passage of warm Atlantic water advection into the Arctic Ocean^84^. It is also characterized by complex hydrography driven by the interaction between the northward-flowing Atlantic waters of the West Spitsbergen Current and the southward flowing Arctic waters of the East Greenland Current that results in strong mesoscale dynamics and intense formation of eddies^85^. Because of the increased transport of warm water by the West Spitsbergen Current (WSC) in recent years, the ice extent in the Svalbard region has been rapidly declining^86^.

### Grouping sampling time into phases according to sea ice position

A value of Open Water Days (OWD) was ascribed to each station of the Baffin Bay campaign, based on how long it had been ice-free before sampling (positive value) or how long after sampling it took to become ice-free (negative value), following the approach applied to the same dataset by Randelhoff et al.^58^. Because OWD correlated clearly with distance to the ice edge (*R* = 0.88), binning and arranging stations according to their OWD enabled us to treat it as the spatial distance relative to the ice coverage.

In Baffin Bay, the dataset was then divided into three phases according to the sea ice conditions based on OWD. The ice-covered period comprised 54 stations that were located under the sea ice (<−10 OWD), in the deeper, Arctic-water-influenced western part of Baffin Bay. The ice break-up period included 24 stations that were within 10 days of the ice break-up (10 days before to 10 days after). The ice-free period included 42 stations that were in open waters for more than 10 days and were located mostly in the eastern shallower part of the Baffin Bay, which is largely influenced by Atlantic water.

Stations in the Fram Strait were divided following a similar time-for-space approach into three different phases determined by ice conditions and timing. The ice-covered phase included 5 stations located in the East Greenland Current domain. Ice break-up phase included 7 stations in the core of the West Spitsbergen Current when it was still the ice edge zone (June 25th to 29th). The ice-free phase included the same stations, but sampled two weeks later (July 6th to 7th), when the ice had disappeared.

The time-for-space approach, although advantageous in terms of data analysis, could involve some caveats, mostly due to the impact of different ocean currents on two sides of the studied areas. In both regions, the differences in the composition of marine snow between eastern and western stations could be amplified by the fact that occupying them two different water mass regimes (Arctic and Atlantic domains) could represent different phenological states of phytoplankton bloom^54,58,71^. To assess this possible bias, the same analysis was performed independently in both water masses. The overall composition of the marine snow was similar, regardless if based on data from two jointed water masses, or only on Atlantic water mass alone (Supplementary Fig. 7).

### Phytoplankton community composition

Relative abundances of distinct algal groups expressed in terms of chl *a* were estimated from in situ pigment measurements processed using the CHEMTAX algorithm^87^. The CHEMTAX software is based on a factorization program that uses “best guess” ratios of accessory pigments to chl *a* for each phytoplankton taxa. These ratios are based on marker pigment concentrations of algal groups that are known to be present in the Arctic Ocean^86^. The methodology of assigning pigment ratios for specific taxa was explained elsewhere^70^. The phytoplankton composition was additionally verified by microscopic identifications^54,55^.

### Underwater vision profiler

Images were collected using the most recent version of the high definition and high-frequency Underwater Vision Profiler (UVP 5hd; Hydroptic, France)^26^. The UVP system detects and counts all objects larger than ~100 µm in a defined and illuminated volume of ~1 L, and automatically stores cut-out vignettes of objects >80 pixels (from approximately 500 µm to a maximum of 200 mm). Particles imaged by the UVP encompass all types of particles from specific ones (e.g., fecal pellets) to very complex ones such as aggregates composed of a mixture of sources (phytoplankton and zooplankton detritus, minerals). For this study, the UVP was mounted onto the carrousel water sampler that also carried a conductivity, temperature, and depth sensor (CTD; SBE 911plus, Seabird Scientific, USA). The acquisition frequency was up to 20 Hz; the carrousel water sampler was lowered with a descent speed of up to 1 m s^−1^.

### Morphological properties of marine snow

The dataset consisted of 24 morphological properties of more than one million images of marine snow. Images were individually classified in the EcoTaxa web application (https://ecotaxa.obs-vlfr.fr/), with the assistance of machine learning classifiers; this classification distinguished non-living (assigned as marine snow) from living (assigned as zooplankton) forms and was fully validated manually. Morphological properties of individual marine snow objects (Supplementary Table 1), representing size (e.g., area, perimeter), shade intensity (e.g., mean/median gray value), shape (e.g., symmetry, elongation), and structural complexity (i.e., homogeneity or heterogeneity, mostly of gray levels) were selected and analyzed.

After trimming the 0.1% most extreme values for each measurement to eliminate outliers and logarithmic transformation of some variables to remove skewness, the combination of those properties was used to build a Principal Component Analysis (PCA) space. PCA analysis was chosen to summarize and hierarchize the morphological information contained in the original multivariate data into few new variables (the principal components) while retaining the interpretability of those components in relation to the original variables. The first 4 components of the PCA space together explained 87% of total variation (Fig. 2). PCA was analyzed via “FactoMineR” and “factoextra” packages in R.

After PCA, each marine snow object was defined by its 4 coordinates in this new morphological space. The k-means clustering method was applied to these PCA coordinates in order to distinguish separate morphotypes of marine snow. K-means classifies objects into a pre-specified number of clusters, such that objects within the same cluster are as similar as possible (i.e., high *intra-class similarity*), whereas objects from different clusters are as dissimilar as possible (i.e., low *inter-class similarity*. Using this method we distinguished five types of marine snow (Fig. 2).

To verify the reproducibility of the method, we defined the morphospace and clusters on (a) Baffin Bay data only, (b) Fram Strait data only, and (c) both (Supplementary Fig. 8); then we classified all objects from both locations according to a, b, and c and compared their cluster membership with Inter-Rater Reliability Analyses (Cohen’s Kappa and agreement test, implemented in the “irr” package in R. The agreement between the classification based on both campaigns and each single campaign dataset was highly significant (99.3%, *k* = 0.991, *p* < 0.001 for the test, when the Baffin Bay was the training set and the Fram Strait dataset was predicted and 92.9% of agreement, *k* = 0.905, *p* < 0.001 when reversed, i.e., Fram Strait as training dataset and the Baffin Bay as predicted). Moreover, we did the same for two predicted datasets, independent from the original, 2-campaigns based classification and again, the agreement of classifying particular particles into their types between various datasets was highly significant (92.2%, *k* = 0.898, *p* < 0.001).

To quantify the morphological diversity of marine snow particles during the various ice phases studied, the Shannon-Wiener diversity index was computed from concentrations in each cluster treated as ‘species’ in the usual formula (package “vegan” in R). Marine snow assemblages dominated by one type would be characterized by low diversity, whereas high diversity corresponds to assemblages where the marine snow is evenly spread among all types. To test the sensitivity of the results to the choice of 5 categories, additional k-means clustering was performed to extract 25, 50, 100 clusters from the morpho-space and the diversity index was recomputed. Conclusions were the same no matter the number of clusters.

The marine snow concentrations were binned over 5-m depth intervals and calculated as the count of particles divided by the water volume sampled (on average 112 L per bin). To compare our approach with more classical descriptions, other indicators of particle dynamics were calculated: total concentration, total equivalent spherical volume, and the slope of the size spectrum. The slope (a) of the particle-size spectrum was calculated from ln(*n*)*ln(a) + b ln(*d*), where *n* is the number of particles in a given size bin and *d* the equivalent spherical diameter of the center of the bin. The vertical attenuation of particle concentration with depth (over the depth range 0-500 m) was quantified by a power law, analogous to the equation used by Martin et al.^63^ to describe flux attenuation, *nz* = n_100_*z^b^/z_100_), where *z* is depth, *z*_100_ is the referenced depth (taken as 100 m) and *n*_z_ is the concentration of particles at depth *z* (*n*_100_ is the concentration at 100 m); the exponent *b* quantifies attenuation with depth. Fluxes could not be calculated because of the lack of information about the density and porosity of the different particle types. However, bulk sinking rates of the five categories were calculated from the deepening of the peak through time^23,51,52,88^.

## Supplementary information

Supplementary Information

## Data Availability

The UVP data that support the findings of this study can be acquired from the corresponding author on request and browsed on EcoTaxa (BaffinBay data: https://ecotaxa.obs-vlfr.fr/prj/149; Fram Strait data: https://ecotaxa.obs-vlfr.fr/prj/257). Background data from Baffin Bay is accessible at the GreenEdge database (http://www.obs.vlfr.fr/proof/php/GREENEDGE/greenedge.php). Background data from Fram Strait is available at the PANGEA database (https://doi.pangaea.de/10.1594/PANGAEA.894874, https://doi.pangaea.de/10.1594/PANGAEA.865180, https://doi.pangaea.de/10.1594/PANGAEA.871949).

## Code availability

The code to build the morphospace from UVP measurements and display examples of images onto is provided at https://github.com/jiho/morphr.
