# Supplementary material for: Marine snow morphology illuminates the evolution of phytoplankton blooms and determines their subsequent vertical export
Source: Nat Commun. 2021 May 14;12:2816. doi: 10.1038/s41467-021-22994-4 (PMC8121919; doi:10.1038/s41467-021-22994-4)
Supplement: Supplementary file 1 — Supplementary Information [file 41467_2021_22994_MOESM1_ESM.pdf]

Marine snow morphology illuminates the evolution of phytoplankton blooms and determines their subsequent vertical export.

Emilia Trudnowska[1], Léo Lacour[2], Mathieu Ardyna[3,4], Andreas Rogge[5,6], Jean Olivier Irisson[4], Anya M. Waite[7], Marcel Babin[2], Lars Stemmann[4]

[1] Institute of Oceanology Polish Academy of Sciences, Sopot, Poland
[2] Takuvik Joint International Laboratory (CNRS and Université Laval), Québec, Qc, Canada
[3] Department of Earth System Science, Stanford University, Stanford, CA, 94305, USA
[4] Sorbonne Université, CNRS, Laboratoire d'Océanographie de Villefranche, LOV, F-06230 Villefranche-sur-Mer, France
[5] Alfred Wegener Institute, Helmholtz Center for Polar and Marine Research, Bremerhaven, Germany
[6] Institute for Ecosystem Research, Kiel University, Kiel, Germany
[7] Ocean Frontier Institute and Dept. of Oceanography, Dalhousie University, Halifax, Nova Scotia, Canada

Corresponding author: emilia@iopan.pl

Abstract

The organic carbon produced in the ocean's surface by phytoplankton is either passed through the food web or exported to the ocean interior as marine snow. The rate and efficiency of such vertical export strongly depend on the size, structure and shape of individual particles, but apart from size, other morphological properties are still not quantitatively monitored. With the growing number of *in situ* imaging technologies , there is now a great possibility to analyze the morphology of individual marine snow. Thus, automated methods for their classification are urgently needed. Consequently, here we present a simple, novel categorization method of marine snow into a few ecologically meaningful functional morphotypes using field data from successive phases of the Arctic phytoplankton bloom. . The proposed approach is a promising tool for future studies aiming to integrate the diversity, composition and morphology of marine snow into our understanding of the biological carbon pump.

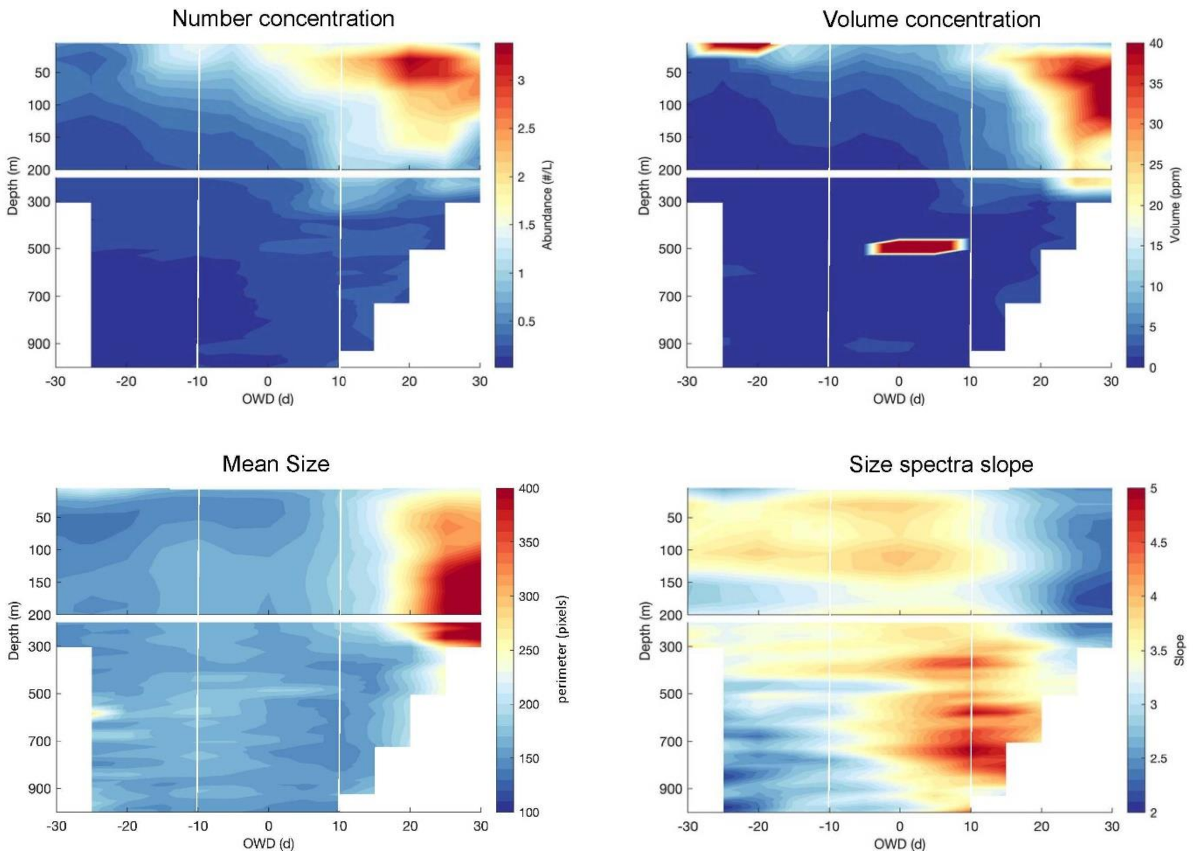

Supplementary Figure 1. Section plots of marine snow total abundance and volumetric particle concentrations, mean size (pixels) and size spectra slopes over depth and ice-related time (open water days (OWD)) in Baffin Bay. Vertical lines indicate the ranges of assigned periods (under ice, ice breakup, ice-free).

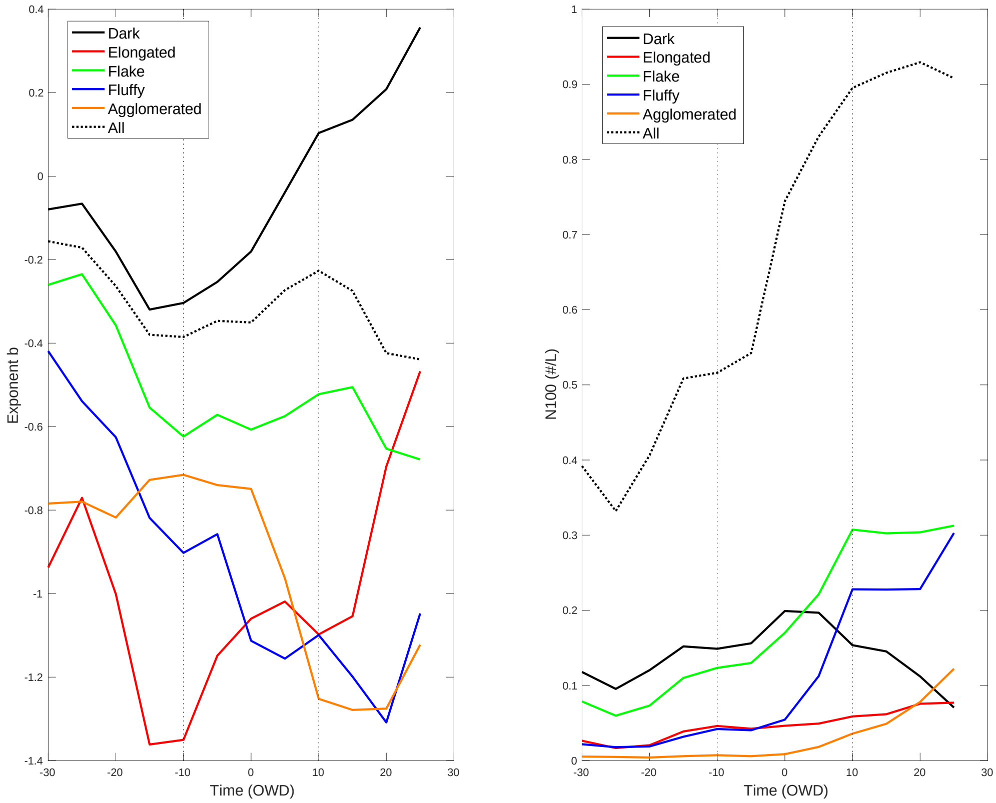

Supplementary Figure 2. Time series of the b value of the exponent of the power law and the abundance at the reference depth of 100m (N100(#/L), fitted on the vertical profiles of abundance of the five categories of marine snow (indicated by colors) and of their sum (All).

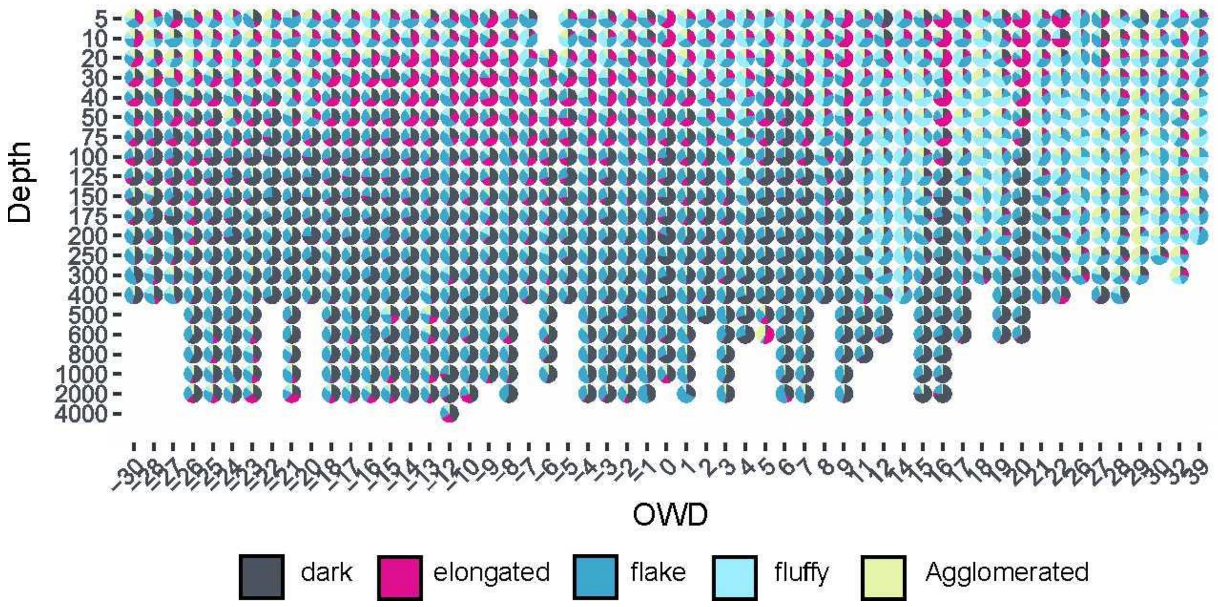

Supplementary Figure 3. The proportions of marine snow morpho-types over depth and ice-related time (open water days (OWD)) in Baffin Bay.

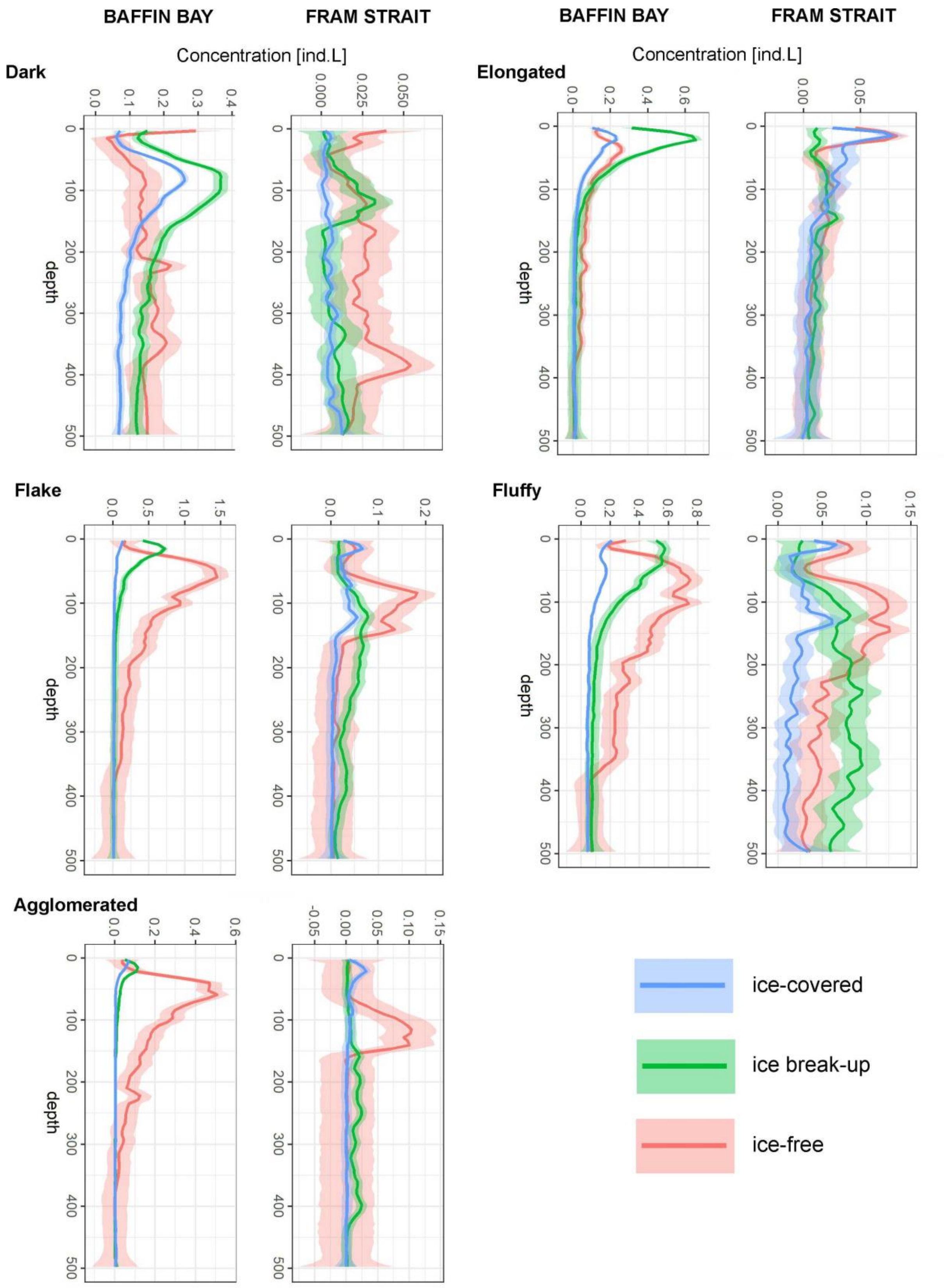

Supplementary Fig. 4. Mean abundances (lines) and standard errors (shades) of marine snow morho-types (dark, elongated, flake, fluffy, agglomerated) in the upper 500 m of the water column in the studied ice-related regimes and locations (marked with colors).

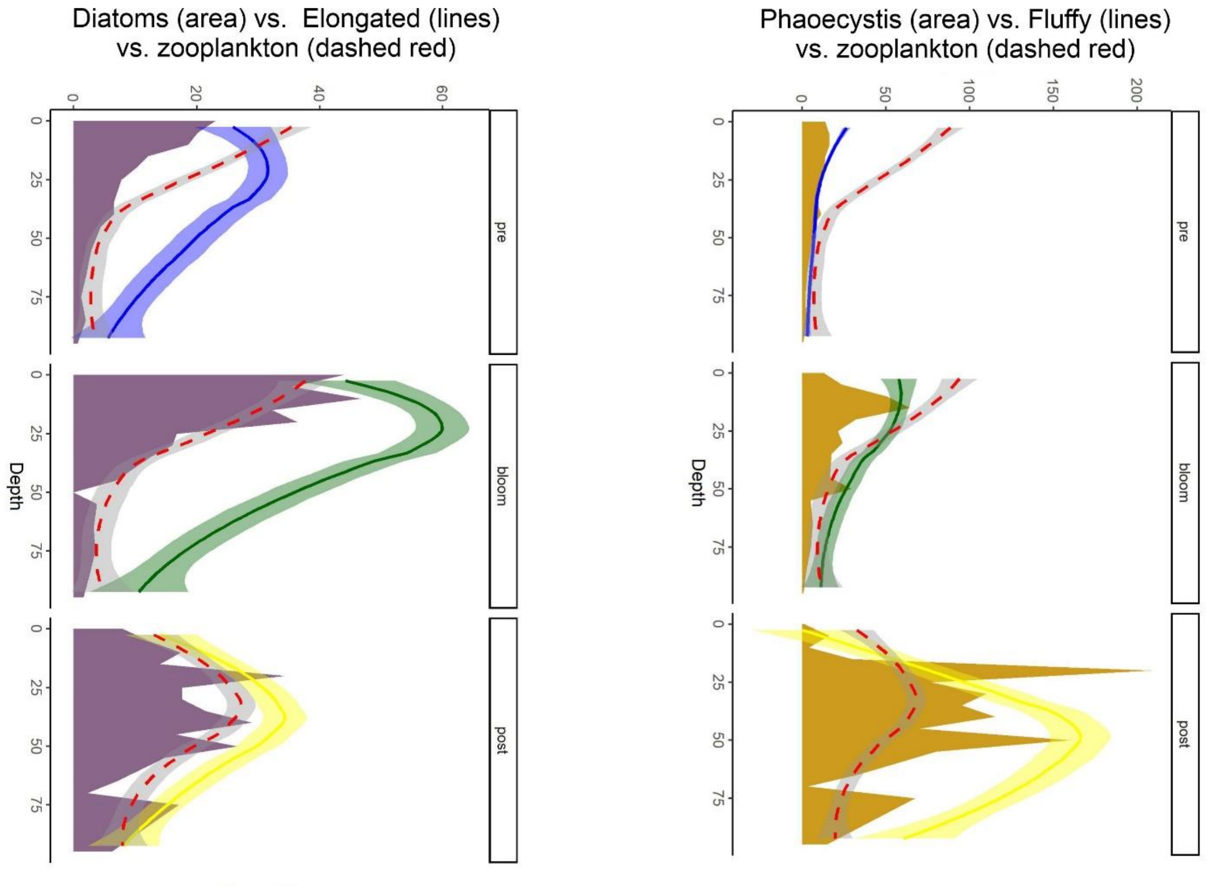

Supplementary Figure 5. Vertical profiles of phytoplankton pigment concentrations (colored areas: Diatoms (purple) and *Phaeocystis* (brownish)), marine snow (elongated and fluffy morphotypes plotted as lines with standard error shades colored according to the bloom phases: pre (ice-covered), bloom (ice break-up), post (ice-free)) in Baffin Bay. Zooplankton abundances are shown as dashed red line.

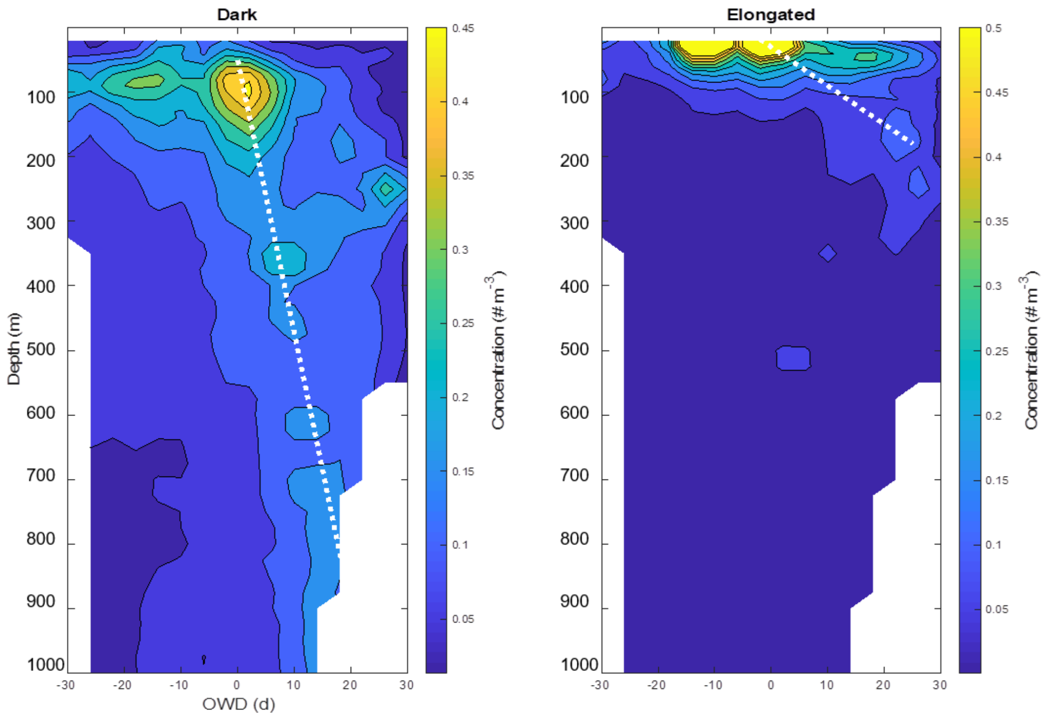

Supplementary Figure 6. The concentrations of dark and elongated morphotypes over depth and ice-related time (open water days (OWD)) in Baffin Bay. White lines show the best linear fit of the maximum abundance of used to calculate the sinking rate. Dark: 38 m.d^{-1} (30.02 - 47.48, 95% confidence intervals). Elongated: 7.2 m.d^{-1} (confident interval of 0.11 - 14.51).

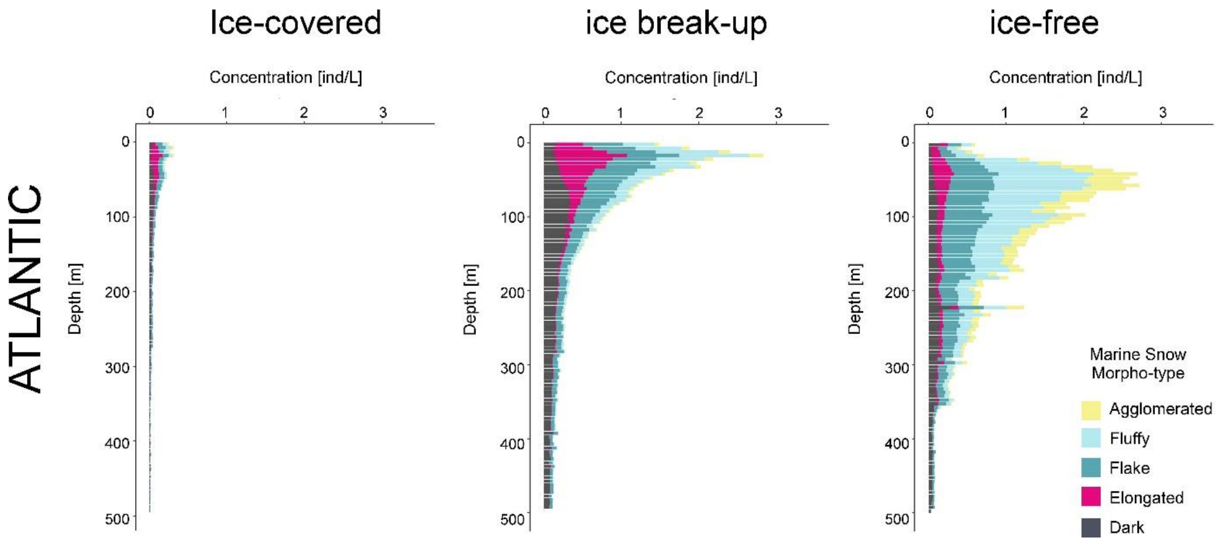

Supplementary Figure 7. Concentrations of marine snow morpho-types over 5-meter depth intervals in defined phases in the Atlantic water masses of the Baffin Bay.

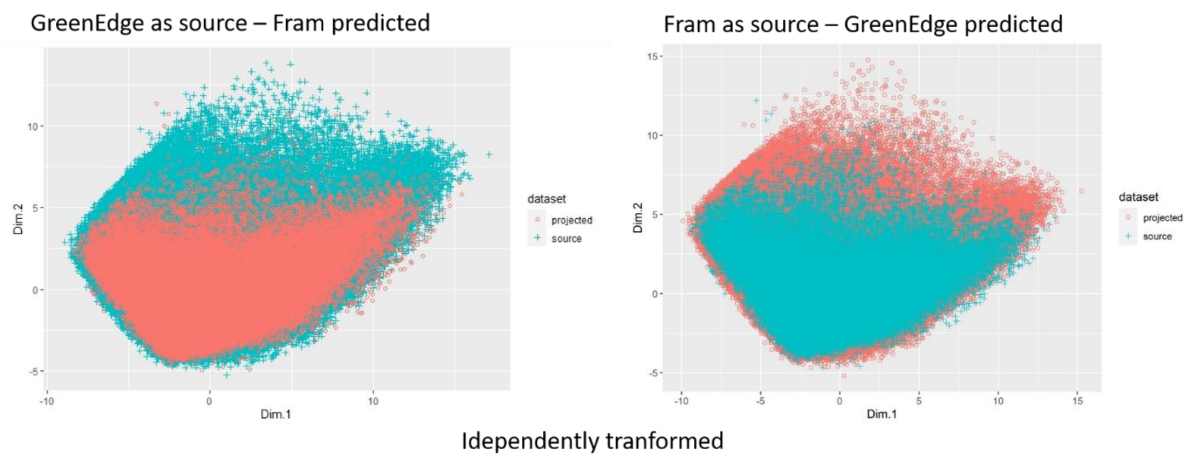

Supplementary Figure 8. The correspondence between PCA morpho-space built on the Baffin Bay dataset (GreenEdge) as source (green colour) and the Fram Strait as projected (orange) and vice versa (right panel).

Supplementary Table. The list of morphological properties, their mean values (given in pixels) for particular morphotypes of marine snow, type, applied transformation and description. Please note that values of the grey level are reversed, i.e., the higher the value the lighter the colour. The ‘trim’ transformation is used to get rid of the extreme variables (falling beyond the 0.001 probability).

Parameter	Dark	Elongated	Mid	Fluffy	Hetero	Type	Transformation	Description
area	2.12	2.14	2.24	2.05	2.72	Size	log10(trim())	Surface area of the object in square pixels
mean	135.9	202.0	229.9	209.2	232.1	Grey level	trim()	Average grey value within the object; sum of the grey values of all pixels in the object divided by the number of pixels
stddev	93.1	50.4	24.9	39.4	25.1	Structure	trim()	Standard deviation of the grey value used to generate the mean grey value
mode	9.4	185.3	245.5	233.2	240.8	Grey level	trim()	Modal grey value within the object
perim	1.68	1.87	1.88	1.67	2.24	Size	log10(trim())	The length of the outside boundary of the object
major	1.21	1.44	1.31	1.19	1.56	Size	log10(trim())	Primary axis of the best fitting ellipse for the object
circ	0.73	0.33	0.41	0.67	0.25	Shape	trim()	circularity : (4∗π∗Area)/Perim2 a value of 1 indicates a perfect circle, a value approaching 0 indicates an increasingly elongated polygon
feret	1.25	1.48	1.38	1.23	1.65	Size	log10(trim())	Maximum feret diameter, i.e. the longest distance between any two points along the object boundary
intden	4.24	4.44	4.61	4.37	5.08	Grey level	log10(trim())	Integrated density. The sum of the grey values of the pixels in the object (i.e. = Area*Mean)
median	146	220	238	220	240	Grey level	trim()	Median grey value within the object.
skew	-0.24	-1.13	-1.83	-0.97	-2.50	Structure	trim()	Skewness of the histogram of grey level values
kurt	-1.35	0.88	4.47	0.48	9.94	Structure	trim()	Kurtosis of the histogram of grey level values
%area	4.61	4.61	4.60	4.61	4.58	Structure	log1p(trim())	Percentage of object's surface area that is comprised of holes, defined as the background grey level
fractal	0.83	0.90	0.92	0.82	1.11	Size	trim()	Fractal dimension of object boundary
skelarea	1.06	1.46	1.56	1.14	2.10	Size	log10(trim())	Surface area of skeleton in pixels. In a binary image, the skeleton is obtained by repeatedly removing pixels from the edges of objects until they are reduced to the width of a single pixel
slope	0.03	0.02	0.03	0.01	0.09	Structure	trim()	Slope of the grey level normalized cumulative histogram
symetrieh	0.32	0.68	0.43	0.34	0.49	Shape	log10(trim())	Bilateral horizontal symmetry index
symetriev	0.32	0.68	0.43	0.34	0.49	Shape	log10(trim())	Bilateral vertical symmetry index
thickr	0.92	1.01	1.09	0.95	1.25	Shape	log1p(trim())	Thickness Ratio; relation between the maximum thickness of an object and the average thickness of the object excluding the maximum
elongation	0.20	0.62	0.26	0.21	0.29	Shape	log10(trim())	The ratio between major/minor axes
range	252.6	183.2	127.0	154.8	154.4	Grey level	trim()	max-min (of grey values)
meanpos	-0.91	-0.40	-0.26	-0.42	-0.19	Structure	trim()	(max−mean)/range (of grey values)
cv	71.3	27.7	11.3	20.0	11.6	Structure	trim()	100∗(stdv/mean) (of grey values)
sr	36.9	26.3	19.6	25.1	16.2	Structure	trim()	100∗(stdv/(max−min)) (of grey values)

Supplementary Table 2. The geographical location and sampling dates of samples stations.

Latitude	Longitude	date	Station ID	Project
68,50	-56,79	09.06.2016	ge_2016_003	Green
68,50	-56,79	09.06.2016	ge_2016_004	Green
68,50	-57,48	10.06.2016	ge_2016_006	Green
68,50	-57,47	10.06.2016	ge_2016_007	Green
68,50	-57,13	10.06.2016	ge_2016_005	Green
68,50	-58,52	11.06.2016	ge_2016_010	Green
68,50	-59,27	11.06.2016	ge_2016_014	Green
68,50	-58,15	11.06.2016	ge_2016_008	Green
68,52	-59,27	11.06.2016	ge_2016_013	Green
68,50	-59,18	11.06.2016	ge_2016_012	Green
68,50	-57,64	11.06.2016	ge_2016_009	Green
68,50	-58,82	11.06.2016	ge_2016_011	Green
68,53	-60,14	12.06.2016	ge_2016_019	Green
68,51	-59,50	12.06.2016	ge_2016_015	Green
68,53	-60,14	12.06.2016	ge_2016_018	Green
68,53	-60,17	12.06.2016	ge_2016_017	Green
68,52	-59,84	12.06.2016	ge_2016_016	Green
68,50	-60,88	13.06.2016	ge_2016_022	Green
68,55	-60,65	13.06.2016	ge_2016_021	Green
68,46	-61,36	13.06.2016	ge_2016_024	Green
68,44	-61,36	13.06.2016	ge_2016_025	Green
68,52	-61,10	13.06.2016	ge_2016_023	Green
68,47	-60,44	13.06.2016	ge_2016_020	Green
68,58	-60,10	14.06.2016	ge_2016_027	Green
68,61	-59,93	14.06.2016	ge_2016_029	Green
68,59	-59,94	14.06.2016	ge_2016_030	Green
68,63	-59,95	14.06.2016	ge_2016_028	Green
68,71	-59,22	15.06.2016	ge_2016_035	Green
68,71	-59,26	15.06.2016	ge_2016_033	Green
68,68	-59,42	15.06.2016	ge_2016_032	Green
68,66	-59,60	15.06.2016	ge_2016_031	Green
68,71	-59,26	15.06.2016	ge_2016_034	Green
68,77	-58,74	16.06.2016	ge_2016_037	Green
68,79	-58,53	16.06.2016	ge_2016_039	Green
68,88	-57,86	16.06.2016	ge_2016_044	Green
68,80	-58,52	16.06.2016	ge_2016_040	Green
68,85	-58,08	16.06.2016	ge_2016_043	Green
68,82	-58,30	16.06.2016	ge_2016_042	Green
68,80	-58,52	16.06.2016	ge_2016_041	Green
68,80	-58,49	16.06.2016	ge_2016_038	Green
68,74	-58,94	16.06.2016	ge_2016_036	Green
69,00	-56,78	17.06.2016	ge_2016_049	Green
69,01	-55,99	17.06.2016	ge_2016_047	Green
68,91	-57,65	17.06.2016	ge_2016_045	Green

69,00	-56,79	17.06.2016	ge_2016_048	Green
68,98	-58,13	17.06.2016	ge_2016_046	Green
69,00	-58,74	18.06.2016	ge_2016_057	Green
69,00	-58,42	18.06.2016	ge_2016_060	Green
69,00	-57,25	18.06.2016	ge_2016_052	Green
69,00	-57,71	18.06.2016	ge_2016_054	Green
69,03	-59,06	19.06.2016	ge_2016_062	Green
68,99	-59,33	19.06.2016	ge_2016_063	Green
69,01	-59,80	19.06.2016	ge_2016_064	Green
69,03	-59,63	19.06.2016	ge_2016_067	Green
69,01	-60,05	20.06.2016	ge_2016_068	Green
69,01	-60,74	20.06.2016	ge_2016_071	Green
69,01	-60,95	20.06.2016	ge_2016_072	Green
69,00	-61,19	21.06.2016	ge_2016_075	Green
69,03	-61,44	21.06.2016	ge_2016_076	Green
69,01	-62,12	21.06.2016	ge_2016_079	Green
68,96	-62,33	21.06.2016	ge_2016_082	Green
69,00	-62,60	22.06.2016	ge_2016_083	Green
68,02	-62,42	24.06.2016	ge_2016_086	Green
68,07	-62,13	25.06.2016	ge_2016_087	Green
68,03	-61,61	25.06.2016	ge_2016_091	Green
68,03	-61,60	25.06.2016	ge_2016_089	Green
68,03	-61,65	25.06.2016	ge_2016_090	Green
68,08	-61,86	25.06.2016	ge_2016_088	Green
68,09	-61,35	25.06.2016	ge_2016_092	Green
68,08	-60,82	26.06.2016	ge_2016_094	Green
68,12	-60,27	26.06.2016	ge_2016_096	Green
68,11	-59,99	26.06.2016	ge_2016_099	Green
68,09	-61,08	26.06.2016	ge_2016_093	Green
68,10	-60,55	26.06.2016	ge_2016_095	Green
68,11	-59,97	26.06.2016	ge_2016_098	Green
68,11	-59,99	26.06.2016	ge_2016_097	Green
68,12	-58,97	27.06.2016	ge_2016_106	Green
68,11	-59,73	27.06.2016	ge_2016_100	Green
68,13	-58,98	27.06.2016	ge_2016_108	Green
68,14	-58,99	27.06.2016	ge_2016_109	Green
68,12	-58,94	27.06.2016	ge_2016_103	Green
68,11	-58,69	27.06.2016	ge_2016_104	Green
68,12	-59,45	27.06.2016	ge_2016_101	Green
68,12	-58,97	27.06.2016	ge_2016_107	Green
68,11	-59,20	27.06.2016	ge_2016_102	Green
68,11	-58,42	27.06.2016	ge_2016_105	Green
68,11	-57,77	28.06.2016	ge_2016_110	Green
68,12	-57,76	28.06.2016	ge_2016_112	Green
68,11	-57,77	28.06.2016	ge_2016_111	Green
68,11	-58,12	28.06.2016	ge_2016_113	Green
70,00	-57,19	29.06.2016	ge_2016_118	Green

70,00	-56,90	29.06.2016	ge_2016_117	Green
68,11	-57,34	29.06.2016	ge_2016_115	Green
68,11	-57,86	29.06.2016	ge_2016_114	Green
68,11	-57,07	29.06.2016	ge_2016_116	Green
70,00	-58,66	30.06.2016	ge_2016_127	Green
70,01	-59,12	30.06.2016	ge_2016_123	Green
70,00	-58,07	30.06.2016	ge_2016_121	Green
70,01	-59,13	30.06.2016	ge_2016_125	Green
70,00	-57,46	30.06.2016	ge_2016_119	Green
70,01	-59,12	30.06.2016	ge_2016_124	Green
70,00	-57,76	30.06.2016	ge_2016_120	Green
70,00	-58,36	30.06.2016	ge_2016_122	Green
70,00	-60,37	01.07.2016	ge_2016_134	Green
70,00	-60,68	01.07.2016	ge_2016_133	Green
70,00	-60,97	01.07.2016	ge_2016_138	Green
70,00	-59,81	01.07.2016	ge_2016_130	Green
70,00	-59,51	01.07.2016	ge_2016_129	Green
70,00	-59,29	01.07.2016	ge_2016_128	Green
70,00	-60,09	01.07.2016	ge_2016_131	Green
70,00	-60,36	01.07.2016	ge_2016_132	Green
69,99	-61,85	02.07.2016	ge_2016_141	Green
70,00	-61,56	02.07.2016	ge_2016_140	Green
70,00	-61,24	02.07.2016	ge_2016_139	Green
70,00	-62,15	02.07.2016	ge_2016_142	Green
70,02	-62,42	02.07.2016	ge_2016_143	Green
70,46	-62,50	04.07.2016	ge_2016_156	Green
70,50	-62,20	04.07.2016	ge_2016_158	Green
70,49	-62,42	04.07.2016	ge_2016_155	Green
70,50	-62,52	04.07.2016	ge_2016_154	Green
70,50	-62,80	04.07.2016	ge_2016_153	Green
70,50	-63,04	04.07.2016	ge_2016_152	Green
70,50	-59,50	05.07.2016	ge_2016_160	Green
70,47	-59,48	05.07.2016	ge_2016_161	Green
70,50	-59,53	05.07.2016	ge_2016_159	Green
70,50	-60,72	06.07.2016	ge_2016_165	Green
70,50	-61,91	06.07.2016	ge_2016_169	Green
70,50	-60,12	06.07.2016	ge_2016_163	Green
70,50	-61,01	06.07.2016	ge_2016_166	Green
70,50	-62,63	06.07.2016	ge_2016_168	Green
70,51	-60,43	06.07.2016	ge_2016_164	Green
70,50	-62,63	06.07.2016	ge_2016_167	Green
70,50	-59,83	06.07.2016	ge_2016_162	Green
69,50	-58,72	07.07.2016	ge_2016_176	Green
70,50	-61,61	07.07.2016	ge_2016_170	Green
70,50	-58,64	07.07.2016	ge_2016_174	Green
70,50	-59,24	07.07.2016	ge_2016_172	Green
70,50	-61,31	07.07.2016	ge_2016_171	Green

69,50	-58,72	07.07.2016	ge_2016_175	Green
70,50	-58,94	07.07.2016	ge_2016_173	Green
69,50	-58,72	07.07.2016	ge_2016_177	Green
69,50	-58,44	08.07.2016	ge_2016_180	Green
69,50	-57,87	08.07.2016	ge_2016_178	Green
69,51	-59,81	08.07.2016	ge_2016_184	Green
69,50	-59,00	08.07.2016	ge_2016_181	Green
69,50	-59,29	08.07.2016	ge_2016_182	Green
69,51	-59,81	08.07.2016	ge_2016_185	Green
69,51	-59,81	08.07.2016	ge_2016_183	Green
69,50	-58,16	08.07.2016	ge_2016_179	Green
69,50	-60,15	09.07.2016	ge_2016_186	Green
69,34	-60,22	09.07.2016	ge_2016_191	Green
69,50	-60,71	09.07.2016	ge_2016_188	Green
69,50	-61,58	09.07.2016	ge_2016_193	Green
69,50	-61,58	09.07.2016	ge_2016_192	Green
69,50	-60,43	09.07.2016	ge_2016_187	Green
69,32	-60,98	09.07.2016	ge_2016_190	Green
69,50	-62,71	10.07.2016	ge_2016_199	Green
69,50	-61,29	10.07.2016	ge_2016_195	Green
69,50	-62,14	10.07.2016	ge_2016_197	Green
69,50	-63,28	10.07.2016	ge_2016_202	Green
69,50	-63,23	10.07.2016	ge_2016_201	Green
77,65	10,28	22.06.2016	ps99_038_2	Fram
77,65	10,28	22.06.2016	ps99_039_1	Fram
78,61	5,06	25.06.2016	ps99_041_1	Fram
78,61	5,05	25.06.2016	ps99_041_6	Fram
79,07	4,18	26.06.2016	ps99_042_1	Fram
78,61	5,03	27.06.2016	ps99_043_3	Fram
79,06	3,47	28.06.2016	ps99_046_1	Fram
79,06	3,58	28.06.2016	ps99_045_1	Fram
79,06	3,28	29.06.2016	ps99_047_1	Fram
78,83	-2,78	29.06.2016	ps99_048_1	Fram
78,82	-2,73	30.06.2016	ps99_048_11	Fram
78,94	-4,65	01.07.2016	ps99_050_1	Fram
78,86	-3,97	01.07.2016	ps99_049_1	Fram
78,99	-5,42	02.07.2016	ps99_051_2	Fram
79,59	5,17	04.07.2016	ps99_054_1	Fram
79,13	2,84	06.07.2016	ps99_059_2	Fram
79,13	4,90	06.07.2016	ps99_057_1	Fram
79,12	2,82	07.07.2016	ps99_059_6	Fram
79,03	6,97	08.07.2016	ps99_062_4	Fram
78,98	9,51	09.07.2016	ps99_064_2	Fram
79,00	8,36	09.07.2016	ps99_063_2	Fram
79,14	6,09	09.07.2016	ps99_066_2	Fram
79,11	4,60	10.07.2016	ps99_069_2	Fram

79,14	6,08	10.07.2016	ps99_066_5	Fram
79,14	6,09	12.07.2016	ps99_074_1	Fram
79,04	4,53	12.07.2016	ps99_075_2	Fram
